# Lymphedema and Elephantiasis of the Lower Limbs: Normalization or Nearly Normalization

**DOI:** 10.7759/cureus.37519

**Published:** 2023-04-13

**Authors:** Jose Maria Pereira de Godoy, Henrique Jose Pereira de Godoy, Ana Carolina Pereira de Godoy, Maria de Fatima Guerreiro Godoy

**Affiliations:** 1 Cardiology and Cardiovascular Surgery, Faculdade de Medicina de São José do Rio Preto (FAMERP), Sao Jose do Rio Preto, BRA; 2 Angiology and Vascular Surgery, Clínica Godoy, Sao Jose do Rio Preto, BRA; 3 General Surgery, Faculdade de Medicina de São José do Rio Preto (FAMERP), São José do Rio Preto, BRA; 4 General Practice, Clínica Godoy, São José do Rio Preto, BRA; 5 Cardiology, Clinica Godoy, Sao Jose do Rio Preto, BRA; 6 Rehabilitation, Faculdade de Medicina de São José do Rio Preto (FAMERP), São José do Rio Preto, BRA; 7 Rehabilitation, Clínica Godoy, São José do Rio Preto, BRA

**Keywords:** edema, normalization, godoy method, treatment, lymphedema

## Abstract

Lymphedema is a clinical condition resulting from a failure in the drainage of the lymphatic system and the consequent formation of edema and is a chronic progressive condition; its development is an active dynamic process. Physiotherapy techniques are the most widely used method for such cases. However, novel concepts and treatment techniques have emerged in recent years. Godoy & Godoy have developed novel therapy concepts proposing the normalization or near normalization of all clinical stages of lymphedema, including elephantiasis.The Godoy & Godoy method has undergone continual evolution, with the improvement of established and the emergence of novel concepts making contributions to the understanding of the causes and treatment of lymphedema. These researchers developed a novel concept of manual lymphatic drainage based on linear movements, cervical lymphatic therapy, a novel concept in mechanical lymphatic drainage, and hand-crafted stockings made with grosgrain material. Therefore, the aim of the present study is to report the new concepts of treatment for lymphedema and the maintenance of such results by the Godoy & Godoy technique in all stages. The Godoy & Godoy method enables the normalization or near normalization of lymphedema in all clinical stages, including elephantiasis.

## Introduction

Lymphedema is a clinical condition that involves the accumulation of molecules in the interstitial space and the consequent retention of liquids. It may be congenital (the individual is born with an abnormal lymphatic system) or acquired. Congenital lymphedema is classified by age: primary congenital lymphedema emerges prior to two years of age; early congenital lymphedema emerges between two and 35 years of age; and late-onset congenital lymphedema emerges after 35 years of age [1,2].

Severity is classified based on volume in comparison to the contralateral limb: < 20% = mild, 20% to 40% = moderate and > 40% = severe. The clinical stage is classified as grade I when the edema emerges throughout the course of the day, grade II when the individual awakens with edema that worsens throughout the course of the day and grade III (also known as elephantiasis), which is more severe than the previous stage and deformities emerge [1-4].

The main forms of treatment are physiotherapy approaches involving manual lymphatic drainage, intermittent pneumatic compression, compression mechanisms with stockings and bandages, exercise and hygiene care [1,2,5]. Pressure therapy may also be included [5]. However, none of these strategies achieves the normalization or near normalization of the edema. The aim of the present study is to report the new concepts of treatment for lymphedema and the maintenance of such results by the Godoy & Godoy technique in all stages.

## Technical report

**Figure 1:**
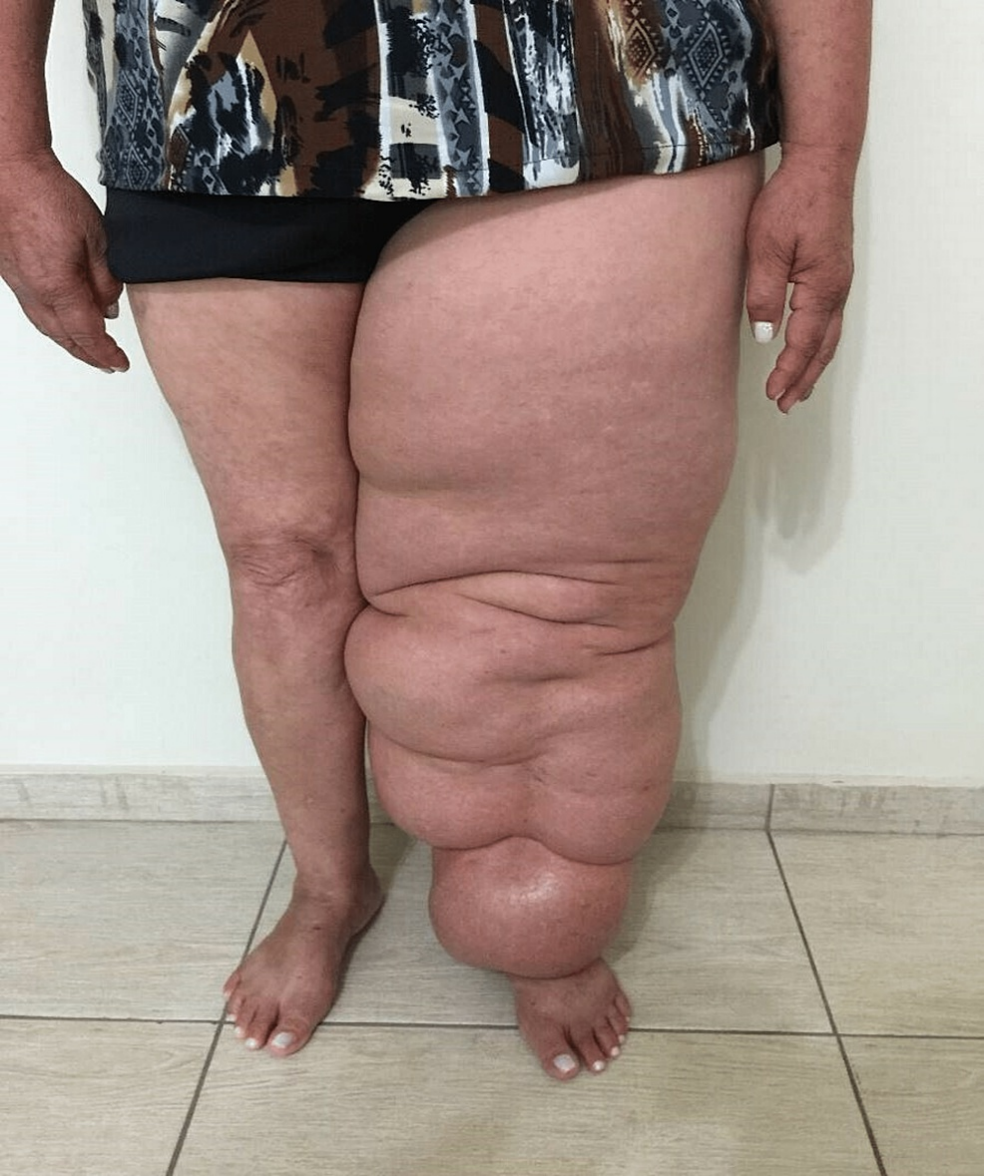
Lymphedema after treatment

**Figure 2:**
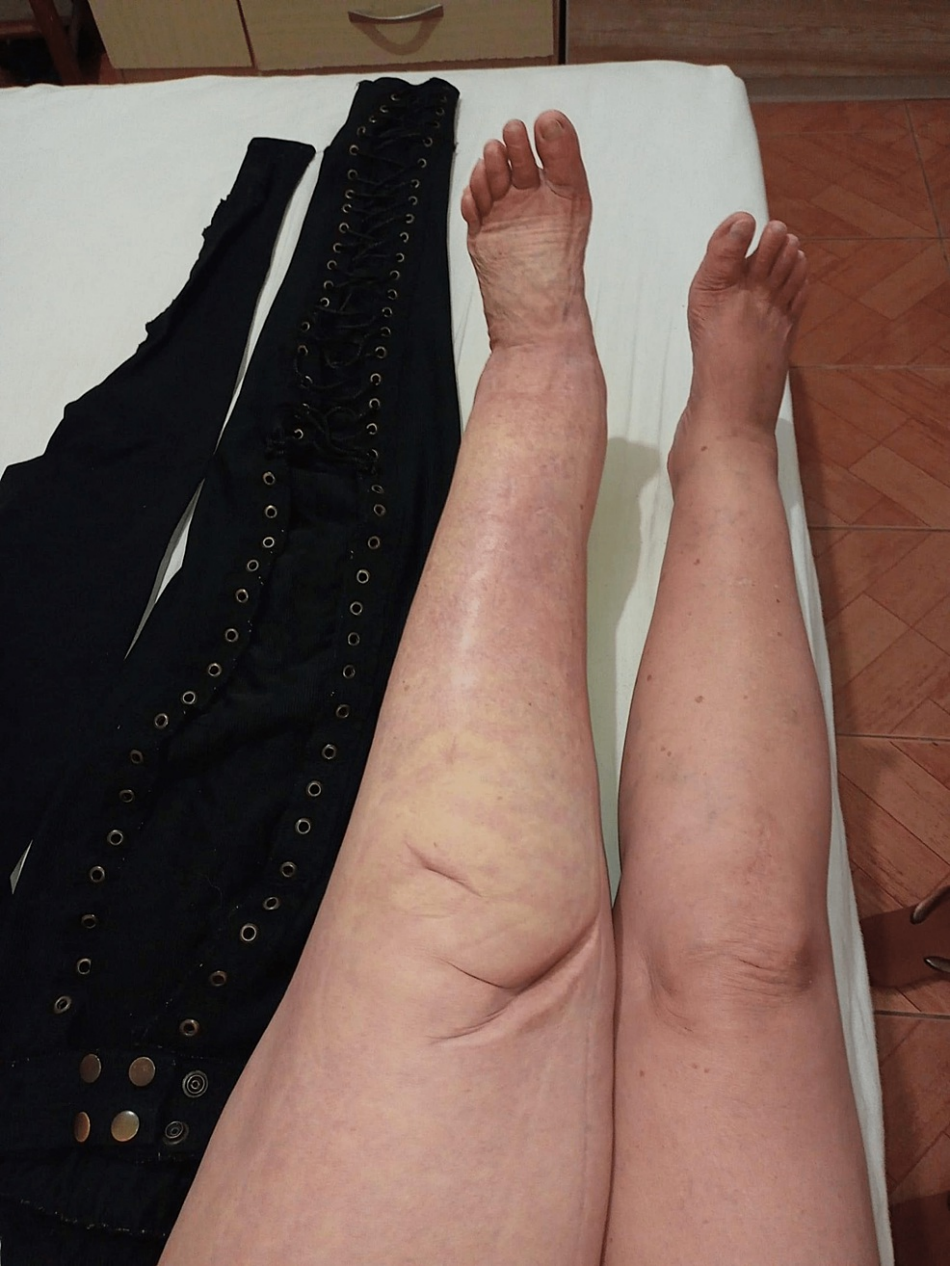
Lymphedema after Godoy method treatment

In recent years, Godoy & Godoy have developed novel therapy concepts proposing the normalization or near normalization of all clinical stages of lymphedema, including elephantiasis (Figures 1, 2) [6-14]. These researchers developed a novel concept of manual lymphatic drainage based on linear movements toward the corresponding lymph nodes [15]. This drainage concept has a scientific basis and can be reproduced in the three stages of scientific investigation: in vitro model, in vivo model and clinic trial [15-20]. Clinical trials involving lymphoscintigraphy have shown the effectiveness of the method both in the formation and drainage of the lymph, and studies have confirmed the reduction in edema [17,18,20].

A novel concept in the stimulation of the lymphatic system was created based on the stimulus of the cervical region, denominated cervical stimuli or cervical lymphatic therapy, which consists of stimuli in the region for 15 to 20 minutes per day. The hypothesis is that this leads to the stimulus of the nervous system, thereby improving lymphatic tone and contraction. Cervical lymphatic therapy is used as a monotherapy for the treatment of lymphedema in children but is indicated for all types of lymphedema [21-28].

A novel concept in mechanical lymphatic drainage was developed based on passive plantar flexion and extension, denominated RAGodoy® (RB Equipamentos Eletrônicos Ltda, Sao Jose do Rio Preto, Brazil) [28-33]. This device has proven to be effective at both the formation and drainage of lymph. Several studies have been conducted over the years and have demonstrated its effectiveness in the treatment of lymphedema. RAGodoy® can be used as a monotherapy, but the concomitant use of compression mechanisms has provided better results. For such, elastic bandages or laced stockings are the most frequently employed [32-34]. Hand-crafted stockings made with grosgrain material have been developed and evaluated over the years and have proved to be effective at reducing edema either alone or in conjunction with another therapy [35-40].

## Discussion

Manual lymphatic therapy, mechanical lymphatic therapy, cervical lymphatic therapy and compression mechanisms are the main components of the Godoy method for the treatment of lower limb lymphedema [6,15,22,29,36]. Hygiene care directed at the prevention of infection is evaluated on an individual basis [41]. If a patient is exposed to the risk of infection, such as a skin injury or mycosis, preventive antibiotic therapy (penicillin benzathine) is indicated during the period of exposure [10]. Psychological, nutritional, family and social support are also indicated, when necessary. The duration of treatment is adapted to each case and depends on the clinical stage of the lymphedema.

In clinical stage I of primary lymphedema, manual lymphatic therapy combined with elastic compression stocking (30/40 mmHg) is sufficient for the normalization of the limb but is combined with cervical lymphatic therapy in all cases. Treatment on a daily basis or every other day is suggested until the normalization of the limb. Intensive therapy for two or more hours a day can also be performed [12,35].

Intensive treatment involves 15 minutes of cervical lymphatic therapy combined with mechanical lymphatic therapy for two to eight hours a day as well as one hour per day of manual lymphatic therapy exclusively on the leg with lymphedema. After normalization, periodic return visits are scheduled as necessary depending on each case, and the patient continues using a ¾ elastic compression stocking (30/40 mmHg). If needed, further lymphatic therapy may be employed to maintain the limb within or near normality [6,12].

In cases of secondary lower limb lymphedema, it is important to determine whether the edema is more distal in the legs or proximal (thighs). When the edema is in the thigh, a half-stocking is normally needed and the best option is grosgrain fabric. If the edema is distal, the procedure is the same as that for primary lymphedema, except that manual lymphatic therapy should be adapted to the physiopathology of each case. In obstructive patterns that lead to lymphatic hypertension, the adapted drainage technique is denominated intermittent compression therapy. For cases without hypertension, we employ the same drainage technique used for primary lymphedema [42].

In primary lymphedema, secondary lesions may occur due to different situations, such as erysipelas, trauma, venous damage, injuries due to inadequate exercise, pressure therapy with high pressure performed in an inadequate manner, inadequate massage techniques, idiopathic cyclic edema, obesity, etc. [43-47]. In such cases of mixed lymphedema, it may be necessary to use half-stockings [12].

For clinical stage II primary lymphedema, intensive treatment is the best option and can reduce the edema by 40% to 70% in one week of treatment performed eight hours per day [7]. This corresponds to eight hours of mechanical lymphatic therapy combined with 15 minutes of cervical lymphatic therapy and one hour of manual lymphatic therapy [6,7]. In cases of mild lymphedema (> 20% increase in volume in comparison to the contralateral side) without fibrosis, it is possible to achieve normalization in one to three weeks. With moderate lymphedema (20% to 40% increase in volume compared to the contralateral side) without fibrosis, two to four weeks may be necessary. For severe lymphedema (> 40% increase in volume compared to the contralateral side), three to six weeks may be needed. In the occurrence of fibrosis, more time may be needed to reach normality. If treatment is only performed one hour a day, normalization takes much longer but is still achievable.

In cases of grade III lymphedema (elephantiasis), intensive treatment is the main option, as it permits a rapid reduction (around 50%) of the edema in one week, which has a positive impact on the patient's quality of life [6,7]. It is possible to normalize or nearly normalize such cases in five to 10 weeks of treatment, depending on the volume of the limb and the occurrence of fibrosis. Normally, two to three weeks of intensive treatment is proposed to achieve 70% to 80% of normalization, followed by maintenance of treatment at home with compression stockings and the RAGodoy® device. The patient returns after one month for a reevaluation and another week of intensive treatment until achieving normalization or near normalization.

The main strategy for the maintenance of the results is the use of elastic stockings for clinical stages I and II of primary lymphedema that have become normalized, possibly alternating with the laced hand-crafted stockings made with grosgrain fabric. In more advanced cases, laced hand-crafted stockings are indicated. Many patients use the RAGodoy® device at home, which assists in maintaining the results [37,38].

The normalization of lymphedema in all clinical stages, including elephantiasis, has been documented in these years and presented at various congresses of the World Society of Lymphology. Currently, evaluations by biopsy before and after treatment show that the method allows stimulating the lysis and synthesis of extracellular matrix proteins, thus changing the characteristics of the skin. The lysis of type I collagen stimulates the production of type III collagen, and elastic fibers are the main changes observed in the first studies, thus bringing a new concept in lymphology [48-50]. This therapeutic evolution changes the concepts of treatment for lymphedema and points to the expectation of a cure for elephantiasis and a drastic reduction in the number of individuals with elephantiasis in the world.

## Conclusions

The Godoy & Godoy method enables the normalization or near normalization of lymphedema in all clinical stages, including elephantiasis. The Godoy method is characterized by constant follow-up of the patients and the results maintenance, in addition to adapting the treatment to each patient's needs and reality.
